# Global and regional brain metabolic scaling and its functional consequences

**DOI:** 10.1186/1741-7007-5-18

**Published:** 2007-05-09

**Authors:** Jan Karbowski

**Affiliations:** 1 Sloan-Swartz Center for Theoretical Neurobiology, Division of Biology 216-76, California Institute of Technology, Pasadena, CA 91125, USA

## Abstract

**Background:**

Information processing in the brain requires large amounts of metabolic energy, the spatial distribution of which is highly heterogeneous, reflecting the complex activity patterns in the mammalian brain.

**Results:**

In this study, it was found, based on empirical data, that despite this heterogeneity, the volume-specific cerebral glucose metabolic rate of many different brain structures scales with brain volume with almost the same exponent: around -0.15. The exception is white matter, the metabolism of which seems to scale with a standard specific exponent of -1/4. The scaling exponents for the total oxygen and glucose consumptions in the brain in relation to its volume are identical, at 0.86 ± 0.03, which is significantly larger than the exponents 3/4 and 2/3 that have been suggested for whole body basal metabolism on body mass.

**Conclusion:**

These findings show explicitly that in mammals: (i) volume-specific scaling exponents of the cerebral energy expenditure in different brain parts are approximately constant (except brain stem structures), and (ii) the total cerebral metabolic exponent against brain volume is greater than the much-cited Kleiber's 3/4 exponent.

The neurophysiological factors that might account for the regional uniformity of the exponents and for the excessive scaling of the total brain metabolism are discussed, along with the relationship between brain metabolic scaling and computation.

## Background

The brain is one of the most energy-expensive tissues in the body [1-3], as it uses large amounts of metabolic energy for information processing [4-7]. Because of this, neural codes are constrained not only by a combination of structural and functional requirements [8-17], but also by energy demands [6,7,18-21]. In general, it has been observed that increased synaptic signaling between neurons leads to higher energy consumption [22], a finding that has been exploited in imaging experiments [23]. Although some theoretical progress has been made in quantifying the contributions of different neurophysiological processes to the total metabolic expenditure of a single neuron [20], the metabolism of large-scale neural circuits has not been investigated quantitatively.

This study attempted to take the first steps in this direction, by studying global and regional *in vivo *brain metabolic scaling. There is a long tradition of applying allometric scaling to problems in biology [1], in particular to whole-body metabolism [1,24], but surprisingly not to cerebral metabolism. The goal of this study was to find, by collecting and analyzing data, the metabolic scaling exponents of different parts of the brain and its global exponent.

It was found that the volume-specific exponents across cerebral regions on brain volume are almost identical, approximately -0.15. Consequently, the energy consumption of the entire brain tissue scales with brain volume, with the exponent of approximately 0.86. The main neurophysiological factors that might cause the increase of the latter exponent well above the putative Kleiber's scaling exponent of 0.75, characterizing whole-body energy expenditure on body mass [24], are identified. The consequences of brain metabolic scaling on its information-processing capacity in the context of brain design are also discussed.

## Results

Oxygen and glucose are the main components involved in the production of adenosine triphosphate (ATP), which is used in cellular energetics [3,4,6], and therefore their rates of utilization provide useful measures of brain metabolism. There are several mammalian species, spanning more than three orders of magnitude in brain size, for which *in vivo *brain metabolic data are available (see Methods).

**Figure 1:**
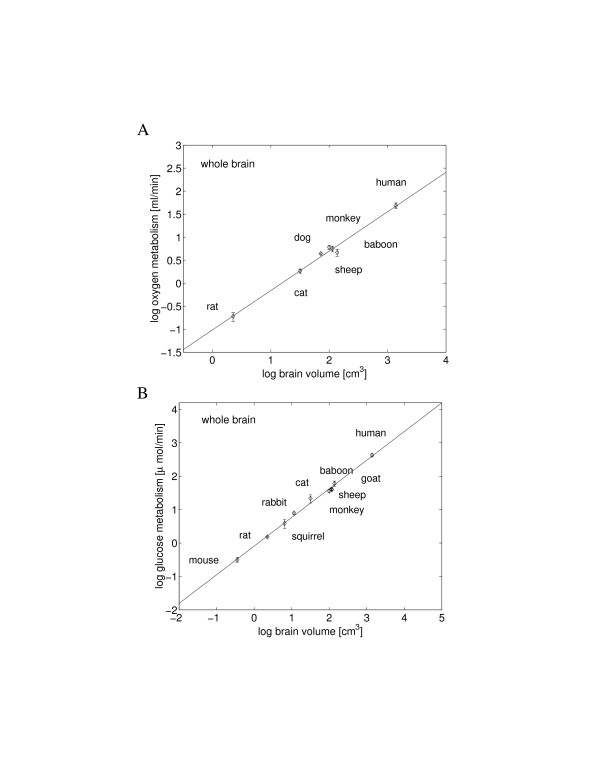
**Scaling of the total basal cerebral metabolism with brain volume**. The least-square fit line for the log – log plot yields the following. (**A**) For the total oxygen consumption rate, the scaling exponent was 0.86 ± 0.04 (y = 0.86x - 1.02, R^2 ^= 0.989, p < 10^-4^, *n *= 7), and its 95% confidence interval was 0.75 to 0.96. (**B**) For the total glucose utilization rate, an identical exponent 0.86 ± 0.03 was found (y = 0.86x - 0.09, R^2 ^= 0.994, p < 10^-4^, *n *= 10) and its 95% confidence interval was 0.80 to 0.91.

The allometric laws characterizing global cerebral metabolism of oxygen and glucose are similar and yield an identical scaling exponent (0.86 ± 0.03) against brain volume (Figure 1). It is important to note that this value is significantly larger (*p *≤ 0.05) than the exponents 0.75 [1,24] and 0.66 [25] found for whole-body mammalian metabolism in relation to body mass.

**Figure 2:**
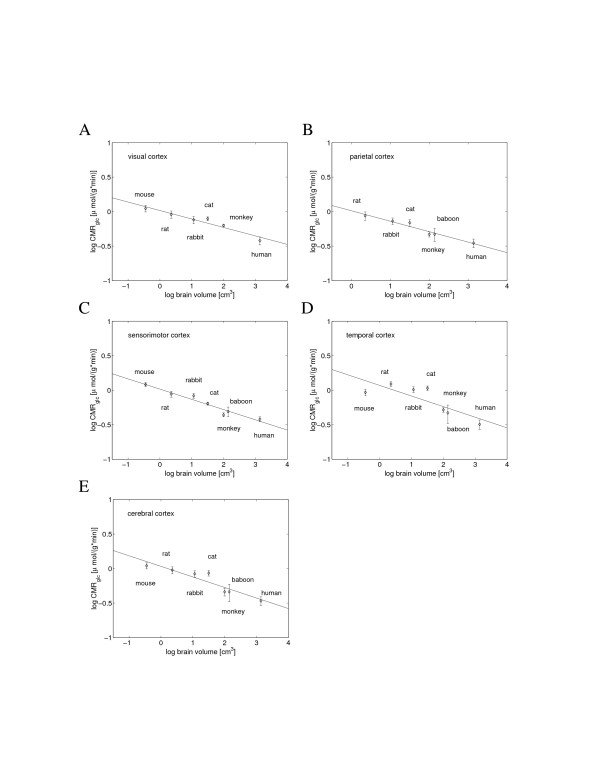
**Scaling of the volume-specific glucose utilization rate, CMRglc, in cerebral cortex with brain volume**. The specific metabolic scaling exponent, corresponding to the slope of the regression line, had the following values: (**A**) -0.12 for visual cortex (y = -0.12x + 0.02); (**B**) -0.15 for parietal cortex (y = -0.15x + 0.01); (**C**) -0.15 for sensorimotor cortex (y = -0.15x + 0.02); (**D**) -0.15 for temporal cortex (y = -0.15x + 0.07). (**E**) Average glucose utilization rate of the entire cerebral cortex yielded the specific exponent -0.15 (y = -0.15x + 0.03).

The cerebral cortex is a critical part of the brain responsible for integrating sensory information, and commanding behavioral and cognitive tasks. Regions of the cerebral cortex differ both in molecular detail and in biological function, which is manifested in a non-uniform distribution of neuronal activity and energy utilization throughout the cortex (Clarke and Sokoloff [5] and Additional file 1). However, despite this heterogeneity, values of the scaling exponents of the regional volume-specific glucose utilization rates on brain volume (CMRglc; glucose cerebral metabolic rate per brain region volume) are surprisingly homogeneous; they are either exactly or close to -0.15 (Figure 2, Table 1). Consequently, the average specific glucose utilization rate of the whole cerebral cortex also scales with brain volume with the exponent -0.15 ± 0.03 (Figure 2E), which is equivalent to the exponent 0.85 ± 0.03 for the metabolism of the entire cortical volume – the value close to that for the whole brain (Figure 1).

**Figure 3:**
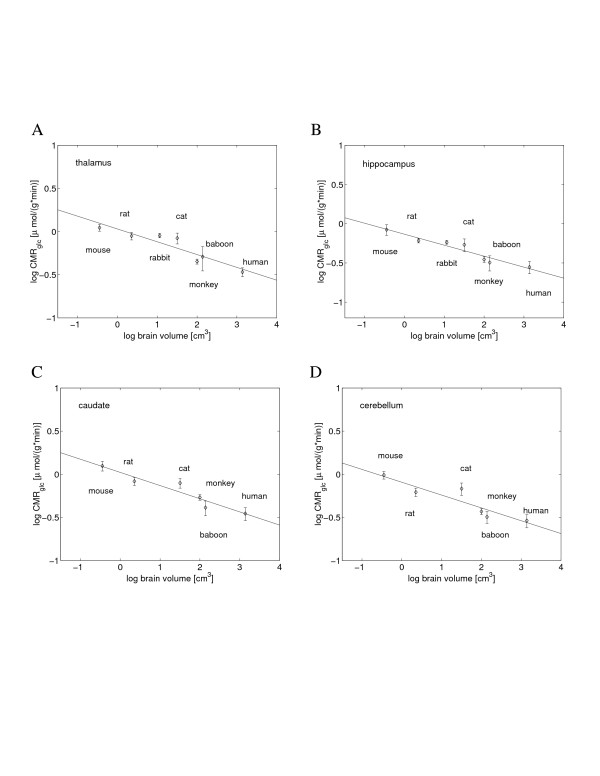
**Scaling of the volume-specific glucose utilization rate in subcortical gray matter with brain volume**. The specific metabolic scaling exponent had the following values: (**A**) -0.15 for thalamus (y = -0.15x + 0.03); (**B**) -0.14 for hippocampus (y = -0.14x - 0.13), which represents limbic structures; (**C**) -0.15 for caudate (y = -0.15x + 0.02), which represents basal ganglia; (**D**) -0.15 for cerebellum (y = -0.15x - 0.09).

Some subcortical structures of gray matter use half of the energy used in the cortex (e.g., limbic structures in cat and monkey; see Clarke and Sokoloff [5] and Additional file 1), yet almost all of them exhibit a similar scaling homogeneity, with metabolic specific exponents also around -0.15 (Figure 3, Table 1). The volume-specific metabolisms of two brain stem structures (the superior colliculus, involved in visual coordination, and the inferior colliculus, involved in auditory processing) seem to be exceptions, as they scale with brain size with the exponent of approximately 0 (Table 1). This might be caused by their highly variable activities (see Additional file 1), and we do not know how other brain stem structures behave. The high degree of allometric uniformity for the most of the subcortical system is even more striking than that for the cortex, as subcortical regions are much more diverse in function and biophysical properties than cortical areas. For example, the thalamus and hippocampus play very different roles in the brain, the former mediating sensory input to the cortex, whereas the latter is implicated in memory processes, yet their scaling exponents and corresponding confidence intervals are almost identical (Figure 3A,B and Table 1).

**Figure 4:**
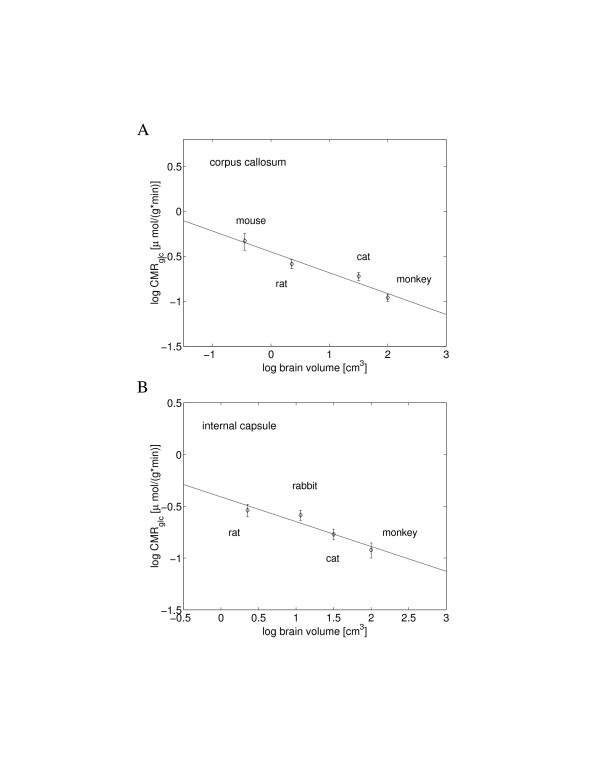
**Scaling of the volume-specific glucose utilization rate in white matter with brain volume**. (**A**) Corpus callosum metabolism yielded the exponent -0.23 (y = -0.23x - 0.45), and (**B**) internal capsule had a similar exponent -0.24 (y = -0.24x - 0.41).

The metabolism of white matter is about three-fold lower than that of gray matter [4,5], and the results in Table 1 indicate that also their scaling exponents might differ. The specific glucose metabolisms of the two main structures of white matter (the corpus callosum and the internal capsule) scale similarly with brain volume with the average exponent, of about -0.25 (Figure 4, Table 1). This value resembles the specific scaling exponent of whole body metabolism against body mass.

## Discussion

The total metabolic exponent 0.86 ± 0.03, found for unanesthetized mammalian brains, implies that cerebral energy use increases more steeply with brain size than does whole-body energy use with body size. This might be a reason why brain size increases slower than its body size, with the exponent 0.75 [26]. However, the metabolic exponent 0.86 undermines Martin's [26,27] well-known argument that the 0.75 scaling of brain size reflects allometric isometry between brain metabolic needs and its size (exponent 1). Instead, the product of the exponents for brain size on body size and brain metabolic rate on brain size gives an exponent for brain metabolic needs on body size of about 0.65, which is lower than the 0.75 expected from the assumption of isometry. Thus, depending on the scaling reference, the total brain metabolism scales either above Kleiber's 0.75 exponent if in relation to brain mass, or below this exponent if in relation to body mass. The discovered uniformity, i.e. constancy, of the cerebral specific exponents in gray matter suggests a common principle underlying the basal metabolism of different brain structures, which might be associated with the homogeneity of synaptic density throughout the gray matter [28,29]. This is covered in more detail later in the discussion.

In analyzing comparative allometry, some authors use phylogenetic approaches to include dependencies in datasets [30,31]. However, these sophisticated methods require as a prerequisite the knowledge of a phylogenetic tree and its associated branching parameters for species of interest. This is not a trivial task to perform, and was therefore not applied in the present analysis. In addition, because correlations in most scaling plots (Figures 1, 2, 3, 4, Table 1) are generally very high, it is likely that taking phylogeny into account would not alter the empirical exponents.

### Key factors in the cerebral metabolic rate

Which factors in the gray matter might account for its total metabolic exponent on brain volume to be >0.75? The likely candidate is the density of glial cells, which provide metabolic support for neurons (including synapses) [32,33]. Two recent studies [34,35] show that the number of glia per neuron increases for larger brains, suggesting that neurons become more energy-expensive with increasing brain size. In particular, the total number of glia in the cerebral cortex of rodents scales with brain size with the exponent 0.89 (Figure 2B in Herculano-Houzel et al [34]), which is close to the empirical metabolic exponent 0.86. It is likely that glia number simply follows the energy demands of neurons, especially their spatial expansion with increasing brain size. Thus, the details of this expansion may provide clues about which neural factors are important.

It has been estimated that neural metabolism is dominated by the Na^+^/K^+^-ATPase ion transport [6,36-38]. The bulk of this comes from active ion fluxes at synapses and along axons (propagation of axon potentials) if the neural firing rate is sufficiently large [20]. The synaptic contribution in a single neuron is proportional to the product of the number of synapses per neuron, the firing rate, release probability, and the postsynaptic charge. The active axon contribution is proportional to its surface area and firing rate. Even when neurons are not firing action potentials, they also consume energy, because of the passive Na^+ ^and K^+ ^ion flow that the electrochemical Na^+^/K^+ ^pump must remove to maintain the gradients of these ions across the membrane [39]. This resting potential contribution is proportional to the total surface area of the neuron. To obtain the total cerebral energy consumption, all three additive contributions must be multiplied by the total number of neurons in the gray matter.

It seems that of all these three neural contributions, the most important for the scaling exponent of total brain metabolism on brain volume is the synaptic contribution. This contribution is proportional to the total number of synapses in the gray matter, which scales with the gray matter volume, and thus brain volume [40,41], with an exponent of 1. The latter occurs because synaptic density is invariant across brain regions and brain size [28,29]. The regional homogeneity of synaptic density correlates with the discovered homogeneity of the regional volume-specific cerebral metabolic scaling (Table 1). Moreover, if the other factors comprising the synaptic contribution (firing rate, release probability, and postsynaptic charge) were independent of brain size, then the synaptically driven total brain metabolism would scale with brain volume with an exponent of 1. As this exponent is, in fact, between 0.75 and 1 (Figure 1), it implies that at least one of these three factors should decrease with brain size. It could be hypothesized that because synaptic sizes and their basic molecular machinery are similar among mammals of different sizes [28,39], the postsynaptic component might be roughly the same among different species. This suggests that the product of the firing rate and release probability should decrease as brains increase in size, with a power of about -0.15, which is in accord with the low firing rates in humans estimated from their basal cerebral metabolic rate [21,42]. This also suggests that the number of active synapses in the background state decreases for bigger brains. That firing rate should decrease with brain (body) size is also consistent with allometric data on the firing rates of avian sensory neurons [43].

The remaining two contributions affecting metabolic rate (the active axons and maintenance of resting potentials) are both proportional to the product of the number of neurons in the gray matter and axonal surface area (assuming that axon surface area constitutes the majority of the neuron's area, especially for bigger brains). Additionally, the active axon contribution is proportional to the average firing rate. Given the above indications that the firing rate probably decreases with brain volume, the active axon contribution becomes less important for the total metabolic exponent as brains increase in size. Thus, one can focus only on the resting potential contribution. Because the product of the number of neurons and axonal surface area is proportional to the ratio of the volume of gray matter to axon diameter (due to the empirical fact that volumes of intracortical axons and gray matter are proportional across species [28]), the resting potential contribution produces the total metabolic exponent that is determined by the scaling exponent of the axon diameter against brain volume. The bigger the axonal exponent, the smaller the metabolic exponent. There are some sketchy experimental indications that axon diameter indeed increases with brain size [44,45], such as in the corpus callosum with the exponent ~0.07 [44]. If similar allometry holds for the gray matter axons, the resting potential contribution would yield a total metabolic exponent also above 3/4.

### Brain metabolism, computation, and design

The fact that the synaptic metabolic contribution decreases with decreasing the rate of release probability, and that the active axon and resting potential metabolic contributions decrease with increasing axon diameter, has interesting functional consequences. A higher failure rate of synaptic transmission as brains get bigger not only saves energy but may also maximize information transfer to postsynaptic neurons [19]. Similarly, increasing axon diameter with brain size accomplishes three functions simultaneously: it reduces the specific metabolic rate, increases the number of synapses per neuron, and increases the speed of signal propagation in cortical circuits, which is proportional to the square root of the axon diameter [46,47]. It is difficult to say at this stage whether these relationships are accidental or are a result of some cerebral optimization.

We can estimate the allometric cost of information processing by determining how the amounts of metabolic energy per neuron and per synapse scale with brain size. As the total energy utilized by the entire cerebral cortex (gray matter) scales with its volume *V*_*g *_with the exponent ~0.85 (Table 1, Figure 2E), the cerebral energy per neuron is proportional to

*V*_*g*_^0.85^/(*ρ*_*n*_*V*_*g*_) ~ *V*_*g*_^0.05 - 0.17^,

that is, it increases with brain size. We used the fact that the neural density, *ρ*_*n*_, scales with brain volume with an exponent between -0.32 and -0.20 (based on data of Haug [48]; see Additional file 1, Figure S2). This increase is in accord with the trend of increasing number of glial cells per neuron in gray matter. The opposite is true for synapses, as the energy per synapse is proportional to

*V*_*g*_^0.85^/(*ρ*_*s*_*V*_*g*_) ~ *V*_*g*_^-0.15^,

where *ρ*_*s *_is the synaptic density (independent of brain size). The finding of an increase in energy expenditure per neuron with increasing brain size is in contrast to findings in liver cells [49], in which metabolism decreases with body mass. This difference reflects the increase of neural size (its wire) and corresponding decrease in density with increasing brain volume (sizes of liver cells are roughly constant [50]). The declining trend for synapses again implies that their active fraction decreases as brains get bigger. These results have implications for coding and cortical organization. The fact that expanding neurons are energetically costly was probably a driving evolutionary force in decreasing their density in larger mammals (Additional file 1, Figure S2) and adopting sparse neural representations [18,20,21]. The latter factor is consistent with the idea of functional modularity of the cerebral cortex [40], that is, that primary information processing takes place in local modules/areas. The sizes of such modules/areas seem to follow scaling rules [12,15,40], and have been shown to have almost brain size-independent connectedness [12] as opposed to neural connectedness, which decays with brain size [11,12].

### Metabolism of gray versus white matter

The data in Table 1 seem to indicate that the metabolic allometries of white matter and gray matter are different. This finding implies that as brains increase in size, the metabolism of white matter is less costly than that of gray matter. This is presumably beneficial for the total cerebral energetic expenditure, as white matter increases disproportionately faster than gray matter [9,40,51]. The difference in metabolism between white and gray matter may be caused by their apparent neuroanatomical differences, as most of the white matter axonal membrane is covered with a myelin sheath, which prevents ion flow and reduces metabolic cost.

### Relation to metabolism of other tissues

Most of the tissues in the body have a much lower specific metabolic rate than the brain, with the exception of four highly active organs: kidney, liver, heart [1] and gut [2]. There are no reliable data on *in vivo *allometric metabolic scaling in these tissues across different species (however, see Wang et al [52], where allometric exponents are given based on only 2–3 species). Allometric *in vitro *studies of Na^+^/K^+^-ATPase in kidney [53] and in brain [54] suggest that these two organs might have comparable specific metabolic exponents. It is possible to estimate and compare metabolic exponents for active tissues indirectly using allometric data on mitochondria size [55], as its total membrane surface area correlates with baseline oxygen consumption [55] (interestingly, the total mitochondrial volume in locomotor muscles is proportional to the maximum metabolic rate in mammals [56]). From these data one can find that the total mitochondrial surface area in brain scales with brain mass with an exponent of 0.86, i.e., exactly as in Figure 1. The corresponding scaling exponents for kidney, liver, and heart against their respective organ masses are smaller and closer to the 0.75 exponent: 0.71, 0.74, and 0.81. These exponents do not seem to relate directly to the exponents of sizes of these tissues on body size, as the masses of kidney and liver increase slower than body mass, while heart mass scales isometrically [1]. Thus, it appears that, in general, higher metabolic exponents do not necessarily lead to lower mass exponents.

If these exponents reflect a real difference in metabolic allometry between cerebral and non-cerebral tissues, it might be caused by differences in membrane chemical composition and ion pump activities, which is known as the "membrane pacemaker theory of metabolism" [38]. For example, it has been shown that heart, liver, kidney, and skeletal muscles display allometric variation in lipid composition, but brain tissue does not [57]. Other potential factors (some of which might be related to the membrane pacemaker hypothesis) affecting differences in the allometries of brain and other tissues include: (i) distinct ways in which energy is used in the brain and in other tissues (e.g., Na^+^/K^+^-ATPase dominates energy consumption only in the brain and kidney [3]); (ii) differences in modes of activity (cells outside nervous system exhibit graded electrical activity without firing Na^+ ^action potentials); (iii) structural differences between brain and other highly active tissues (the size of non-cerebral cells is virtually independent of body mass and the cells lack elongated processes with synapses [50]); (iv) the existence of the blood-brain barrier, which restricts direct transport of molecules between bloodstream and nerve cells [39], and may affect substrate utilization rate and/or neural activity [58,59].

### Supply-limited models of metabolic scaling

In many studies of whole-body metabolism, the scaling exponent 0.75 was found [1,24], and it was argued that this value follows from a general model of hierarchical fractal-like transport networks [60], or from constrained geometric networks with balanced supply and demand [61]. Both of these models are based on the assumption that metabolic rates are determined solely by resource supply rates and are independent of cellular energy expenditure. This single-cause assumption has been challenged recently [62,63]. The main arguments against the supply-limited models are that: (i) rate of blood flow adjusts itself to the physiological demands of tissue, and in resting animals is well below its maximal limit; and (ii) cellular metabolic rates decline with increasing body size [49] (at least for non-cerebral cells). Thus, a "multiple-causes" scenario of metabolic scaling has been proposed [62,63], which in essence argues that supply rate is only one of the factors and should be considered together with other factors characterizing utilization of cellular energy. The approach taken in this paper is similar in spirit, i.e., given that the total cerebral metabolic exponent is 0.86, simple supply-limited models are rejected as a possible explanation for brain metabolic scaling. Instead, the most energetically expensive cellular factors that are most likely to affect the metabolic exponent were identified. In this sense, this approach can also be viewed as a multiple-cause model of cerebral metabolic scaling.

## Conclusion

Figures 1, 2, 3 and Table 1 provide empirical evidence that the scaling exponents describing global and regional brain metabolism are significantly different (*p *≤ 0.05) from the much-cited exponent of 3/4, which calls into question the direct applicability of supply-limited models [60,61] to brain metabolism. The exceptions are white matter structures, which seem to exhibit "regular" metabolic exponents (Figure 4). The empirical results presented in this paper show a striking uniformity of the allometric metabolic exponents over almost the entire gray matter of mammalian brains at normal resting conditions, despite anatomical and functional heterogeneity of different regions and their different levels of activation. This regional scaling uniformity is surprising, as activity level could potentially affect the scaling exponent. For example, the total metabolic rate of maximally exercised body scales with body mass well above 0.75, with an exponent of 0.88 [56,64].

## Methods

I*n vivo *data of the cerebral oxygen (CMRO_2_) and glucose (CMRglc) utilization rates of unanesthetized adult animals at resting conditions were collected from various sources [5,65-99] (see Additional file 1). In those studies, the measurements of glucose utilization in all species were performed by essentially the same method or its modification (in human and baboon), and thus all glucose data are directly comparable. There is a small method variability for oxygen data, as the same technique was applied to five of seven species (except cat and dog). However, this variability does not affect the scaling exponent (Figure 1A); it is the same even if only single-method mammals are included in the plot. Glucose utilization data represent both global and regional cerebral metabolism.

The investigated mammals comprised: Swiss mouse, squirrel, rabbit, goat (all glucose data only), dog (oxygen data only), cat, Sprague-Dawley rat, macaque monkey, baboon, sheep, and human. The investigated brain structures comprised: cerebral cortex (visual, prefrontal, frontal, sensorimotor, parietal, temporal, cingulate, occipital), thalamus (including lateral geniculate nucleus and medial geniculate nucleus), hypothalamus (and separately, the mammillary body), cerebellum (including cerebellar cortex and dentate nucleus), basal ganglia (caudate, substantia nigra, globus pallidus), limbic system (hippocampus, amygdala, septum), brain stem (superior colliculus, inferior colliculus), and white matter (corpus callosum, internal capsule). In cases when there was more than one data point for a given animal or a brain structure, an arithmetic mean of all values was taken.

Allometric metabolism of the entire cerebral cortex (presented in Figure 2E), was obtained by computing an arithmetic mean of glucose utilization in the eight cortical areas listed above for each animal. Glucose utilization of a given area was itself an average of values taken from different sources. If data for all eight areas were not available, data were averaged over fewer areas. For consistency, an alternative method of averaging was also used: the first averaging was performed for a given data source, and the second for different sources representing the same animal. In this method, because different sources differed in the number of cortical areas studied, averaging in many cases was performed over a significantly different number of areas. However, both methods gave statistically identical scaling exponents for the cerebral cortex metabolism (Additional file 1, Figure S1).

The allometric metabolism of the entire brain was obtained by using either direct data quoted by authors, or, if not available, by computing an arithmetic mean of glucose consumption in all brain structures provided by the authors. For all scaling plots, the brain volumes were taken from Hofman [41] and Stephan et al [100], or from the source.

## Supplementary Material

Additional file 1 **"Supplementary information for 'Global and regional brain metabolic scaling...'"**. The file contains figures with the scaling of neural density on brain size. It also contains tables with collected data of glucose (and oxygen) metabolic utilization rates in various brain regions across mammalian species. Glucose utilization data were taken from the following sources for: mouse [65,66]; rat [67-73]; rabbit [74]; squirrel [75]; cat [76-80]; macaque monkey [81-84]; baboon [85]; goat [86]; sheep [87] (both glucose and oxygen); human [5,88-93]. Oxygen consumption data were taken from the following sources for: rat [94]; cat [95]; dog [96]; macaque monkey [97]; baboon [98]; human [5,99]. Click here for file

## Figures and Tables

**Table 1:** Specific scaling exponents of the regional cerebral glucose utilization rate CMRglc against brain volume

**Brain structure**	**Scaling exponent**	**95% confidence intervals**	**Correlation R^2^**	***p*-value**	**Species (*n*)**
**Cerebral cortex**	-0.15 ± 0.03	-0.22 to -0.08	0.870	0.0022	7
Visual	-0.12 ± 0.03	-0.17 to -0.08	0.932	0.0018	6
Prefrontal	-0.17 ± 0.03	-0.23 to -0.10	0.953	0.0044	5
Frontal	-0.14 ± 0.02	-0.17 to -0.12	0.997	0.0015	4
Sensorimotor	-0.15 ± 0.02	-0.19 to -0.11	0.945	0.0002	7
Parietal	-0.15 ± 0.03	-0.20 to -0.11	0.954	0.0008	6
Temporal	-0.15 ± 0.05	-0.27 to -0.03	0.680	0.0224	7
Cingulate	-0.16 ± 0.03	-0.23 to -0.09	0.912	0.0030	6
Occipital	-0.20 ± 0.12	-0.53 to 0.12	0.563	0.1439	5
**Thalamus**	-0.15 ± 0.03	-0.22 to -0.08	0.858	0.0027	7
**Hypothalamus**	-0.10 ± 0.04	-0.20 to -0.01	0.692	0.0402	6
Mammillary body	-0.15 ± 0.07	-0.30 to -0.00	0.773	0.0495	5
**Cerebellum**	-0.15 ± 0.04	-0.24 to -0.06	0.840	0.0102	6
**Basal ganglia**					
Caudate	-0.15 ± 0.03	-0.22 to -0.08	0.905	0.0035	6
Substantia nigra	-0.14 ± 0.02	-0.18 to -0.10	0.956	0.0007	6
Globus pallidus	-0.16 ± 0.04	-0.24 to -0.08	0.926	0.0088	5
**Limbic system**					
Hippocampus	-0.14 ± 0.03	-0.19 to -0.09	0.919	0.0007	7
Amygdala	-0.12 ± 0.03	-0.17 to -0.07	0.919	0.0025	6
Septum	-0.16 ± 0.03	-0.23 to -0.09	0.944	0.0057	5
**Brain stem**					
Superior colliculus	-0.06 ± 0.05	-0.19 to 0.06	0.448	0.2168	5
Inferior colliculus	0.05 ± 0.09	-0.14 to 0.23	0.174	0.4843	5
**White matter**					
Corpus callosum	-0.23 ± 0.09	-0.40 to -0.06	0.947	0.0271	4
Internal capsule	-0.24 ± 0.12	-0.48 to 0.00	0.902	0.0504	4

The *t*-test showed that population mean of the exponents in the second column was significantly greater than the exponent -0.25 (*p *= 9 × 10^-9^, degrees of freedom (df) = 21 if white matter included; *p *= 2 × 10^-9^, df = 19 if white matter excluded).
