# Supplementary material for: Global and regional brain metabolic scaling and its functional consequences
Source: BMC Biol. 2007 May 9;5:18. doi: 10.1186/1741-7007-5-18 (PMC1884139; doi:10.1186/1741-7007-5-18)
Supplement: Additional file 1 — "Supplementary information for 'Global and regional brain metabolic scaling...'". The file contains figures with the scaling of neural density on brain size. It also contains tables with collected data of glucose (and oxygen) metabolic utilization rates in various brain regions across mammalian species. Glucose utilization data were taken from the following sources for: mouse [65,66]; rat [67-73]; rabbit [74]; squirrel [75]; cat [76-80]; macaque monkey [81-84]; baboon [85]; goat [86]; sheep [87] (both glucose and oxygen); human [5,88-93]. Oxygen consumption data were taken from the following sources for: rat [94]; cat [95]; dog [96]; macaque monkey [97]; baboon [98]; human [5,99]. [file 1741-7007-5-18-S1.pdf]

Supplementary information for

"Global and regional brain metabolic scaling and its functional consequences"

Jan Karbowski

Sloan-Swartz Center for Theoretical Neurobiology,
Division of Biology 216-76,
California Institute of Technology, Pasadena, CA 91125, USA

1. **Supplementary Figures.**

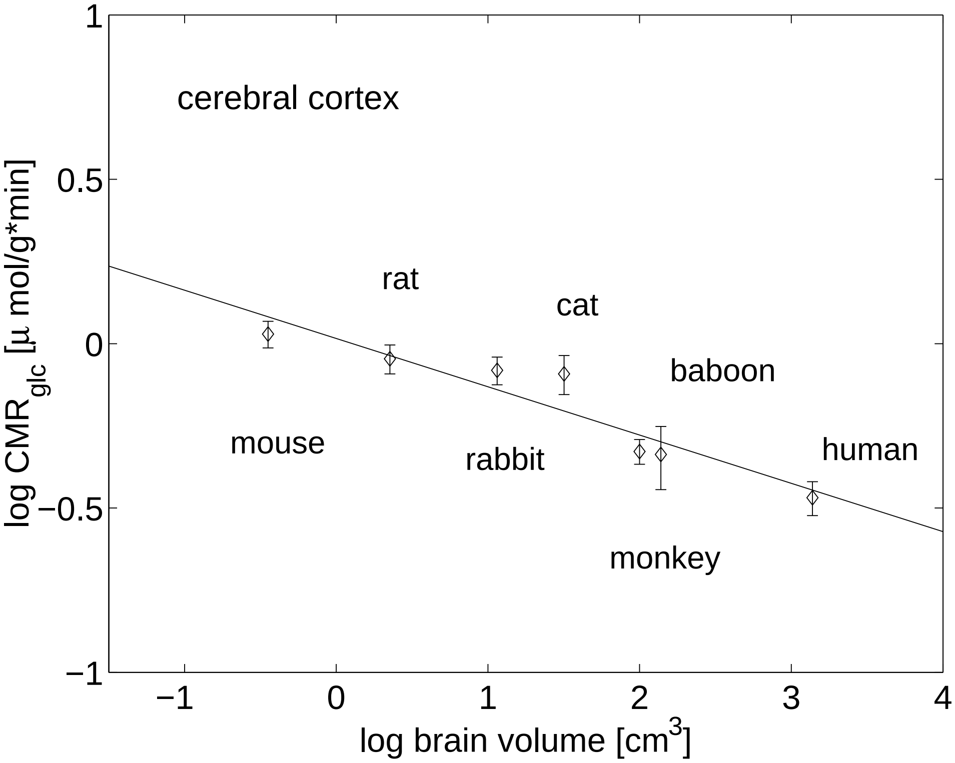

Figure S1. Allometric scaling of the average glucose utilization in the cerebral cortex with brain volume. The scaling exponent is -0.15 ± 0.02 (R^2= 0.89, p= 0.0015) and its 95% confidence interval is $(-0.21, -0.09)$. Thus, the exponent is statistically identical to that in Fig. 2E in the main text, although the methods of averaging differ.

For the purpose of the discussion in the main text regarding neural function, we also determine the scaling of the neural density ρ_n with the brain volume V_b, which has been a subject of some controversy recently. The older data by Tower (1954) indicated that $\rho_n \propto V_b^{-0.32}$. The later data by Rockel et al. (1980) indicated that the surface density of neurons was brain size independent, which suggests that ρ_n should scale with V_b the same way as does the inverse of cortical thickness. Since the latter scales with brain volume with the exponent ≈ 0.10 (Schlenska, 1974; Prothero, 1997; Hofman, 1988), the Rockel et al. (1980) data imply that $\rho_n \propto V_b^{-0.10}$. However, both of these results may suffer from a small sample of animals used (11 in Tower, and only 5 in Rockel). Additionally, Rockel et al. (1980) counted the number of neuronal cell bodies in a narrow strip (≈ 30 μm) through the depth of the neocortex, which does not take into account horizontal spacing between neurons and may be the source of discrepancy between the two results.

In Fig. S2, volume and surface densities of neurons in the gray matter are plotted as a function of brain volume for a larger number of animals, using more recent data of Haug (taken from Figs. 7 and 13 in Haug, 1987). Despite scattered data points there exist significant correlations between both densities and brain volume, and the scaling exponents are, surprisingly, statistically identical ≈ -0.20. The scaling exponent for the surface density is compatible with the result of Tower (1954), if we take the cortical thickness scaling into account, i.e., in this case $\rho_n \propto V_b^{-0.32}$. On the other hand, the scaling exponent for the volume density ρ_n is exactly in the middle of the values obtained by Tower (1954) and implicated by Rockel et al. (1980), i.e., $\rho_n \propto V_b^{-0.20\pm0.03}$. We conclude that neural density decreases with increasing brain volume with the allometric exponent in the range between -0.32 and -0.20.

Tower DB (1954) Structural and functional organization of mammalian cerebral cortex: the correlation of neurone density with brain size. *J. Comp. Neurol.* **101**: 19-52.

Rockel AJ, Hiorns RW, Powell TPS (1980) The basic uniformity in structure of the neocortex. *Brain* **103**: 221-244.

Haug H (1987) Brain sizes, surfaces, and neuronal sizes of the cortex cerebri: A stereological investigation of Man and his variability and a comparison with some mammals (primates, whales, marsupials, insectivores, and one elephant). *Am. J. Anatomy* **180**: 126-142.

A

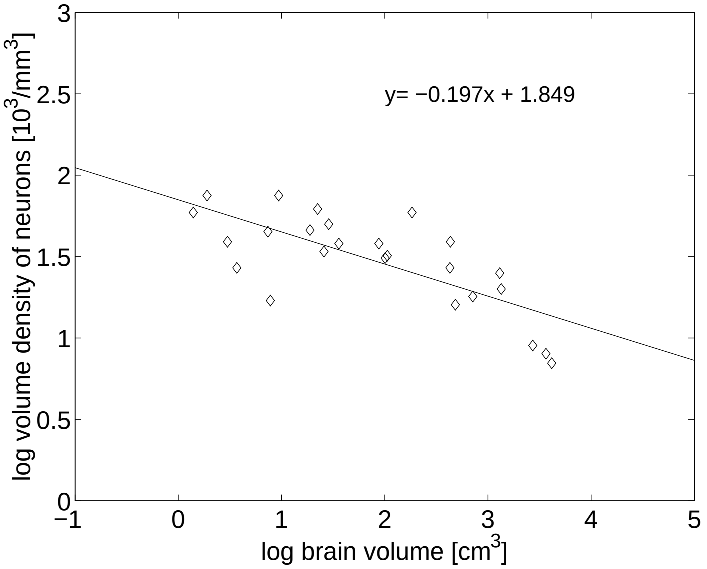

B

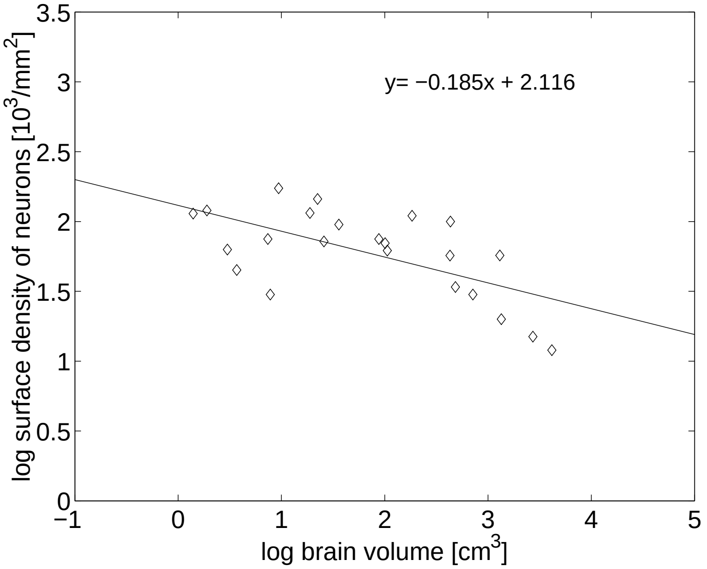

Figure S2. Scaling of the cortical neuronal densities with brain volume. The data are taken from Haug (1987) and re-plotted in a log-log scale. (A) Volume density scales with brain volume with the exponent -0.20 ± 0.03 ($y = -0.20x + 1.85$, $R^2 = 0.540$, $p < 10^{-4}$, $N = 25$). The 95% confidence interval for the scaling exponent is $(-0.28, -0.12)$. (B) Surface density scales with brain volume with the

exponent -0.19 ± 0.04 ($y = -0.19x + 2.12$, $R^2 = 0.392$, $p = 0.0014$, $N = 23$). The 95% confidence interval for the scaling exponent is $(-0.29, -0.08)$. Note that both exponents are almost identical.

2. **Supplementary Tables.**

Table S1.
Brain oxygen consumption data in adult awake unanesthetized animals.

Species	Oxygen consumption $(ml/g \cdot min)$	Total oxygen consumption (ml/min)	Reference
rat	0.084 ± 0.019	0.190	Linde et al 1999
cat	0.058 ± 0.006	1.844	Stingele et al 1996
dog	0.061 ± 0.002	4.392	Nakanishi et al 1997
monkey	0.060 ± 0.005	6.000	Nemoto et al 1994
sheep	0.049 ± 0.007	5.586	Pell et al 1983
baboon	0.034 ± 0.006	4.658	Sahlin et al 1987
human	0.035 ± 0.005	48.615	Clarke et al 1994
			Madsen et al 1991

Table S2.
Brain glucose utilization data in adult awake unanesthetized animals.

Species	Glucose utilization $(\mu mol/g \cdot min)$	Total glucose utilization $(\mu mol/min)$	Reference
mouse	0.89 ± 0.14	0.32	Bouilleret et al 2000
rat	0.68 ± 0.04	1.52	Nehlig et al 1988
			Waschke et al 1993
			Levant et al 2004
squirrel	0.60 ± 0.18	3.88	Frerichs et al 1995
rabbit	0.69 ± 0.05	7.93	Passero et al 1981
cat	0.69 ± 0.19	21.78	Chugani et al 1991
			Sokoloff 1984
monkey	0.36 ± 0.03	35.98	Kennedy et al 1978
sheep	0.35 ± 0.03	40.18	Pell et al 1983
goat	0.35 ± 0.04	40.09	Pelligrino et al 1987
baboon	0.44 ± 0.08	60.40	Meguro et al 1999
human	0.31 ± 0.03	428.55	Clarke et al 1994

Table S3.
Visual cortex glucose utilization rate in mammals.

Species	Glucose utilization ($\mu mol/g \cdot min$)	Reference
mouse	1.11 ± 0.12	Quelven et al 2004, estim.
rat	0.86 ± 0.14	Waschke et al 1993
rat	0.82 ± 0.12	Dube et al 2001
rat	0.97 ± 0.05	Nehling et al 1988
rat	1.00 ± 0.10	Nehling et al 2000
average rat	0.91 ± 0.11	
rabbit	0.76 ± 0.09	Passero et al 1981
cat	0.52 ± 0.03	Herdman et al 1989
cat	0.95 ± 0.09	Sokoloff 1984
cat	0.89 ± 0.03	Schwartzman et al 1986
average cat	0.79 ± 0.05	
monkey	0.59 ± 0.02	Kennedy et al 1978
monkey	0.66 ± 0.02	Palombo et al 1990
average monkey	0.63 ± 0.02	
human	0.39 ± 0.05	De Volder et al 1997
human	0.37 ± 0.04	Heiss et al 1985
average human	0.38 ± 0.05	

Table S4.
Prefrontal cortex glucose utilization rate in mammals

Species	Glucose utilization ($\mu mol/g \cdot min$)	Reference
mouse	1.22 ± 0.12	Bouilleret et al 2000
rat	0.99 ± 0.15	Dube et al 2001
rat	1.03 ± 0.05	Nehling et al 1988
rat	0.91 ± 0.21	Zocchi et al 2001
average rat	0.98 ± 0.14	
monkey	0.40 ± 0.03	Porrino et al 2002
baboon	0.52 ± 0.14	Meguro et al 1999
human	0.33 ± 0.04	Blin et al 1991

Table S5.

Frontal cortex glucose utilization rate in mammals.

Species	Glucose utilization ($\mu mol/g \cdot min$)	Reference
mouse	1.07 ± 0.10	Bouilleret et al 2000
rat	0.84 ± 0.16	Dube et al 2001
rat	0.86 ± 0.04	Levant et al 2004
rat	0.78 ± 0.09	Waschke et al 1993
average rat	0.83 ± 0.10	
monkey	0.34 ± 0.08	Noda et al 2002
monkey	0.58 ± 0.02	Palombo et al 1990
average monkey	0.46 ± 0.05	
human	0.37 ± 0.09	De Volder et al 1997
human	0.28 ± 0.02	Redies et al 1989
human	0.25 ± 0.01	Blomqvist et al 1998
human	0.41 ± 0.02	Heiss et al 1985
human	0.33 ± 0.06	Maquet et al 1992
human	0.41 ± 0.12	Haier et al 1992
average human	0.34 ± 0.06	

Table S6.

Cingulate cortex glucose utilization rate in mammals.

Species	Glucose utilization ($\mu mol/g \cdot min$)	Reference
mouse	1.06 ± 0.12	Quelven et al 2004, estim.
rat	0.91 ± 0.16	Dube et al 2001
rat	1.21 ± 0.05	Levant et al 2004
rat	0.83 ± 0.09	Blin et al 1991
average rat	0.98 ± 0.10	
cat	0.70 ± 0.06	Schwartzman et al 1986
monkey	0.37 ± 0.09	Noda et al 2002
monkey	0.40 ± 0.02	Palombo et al 1990
average monkey	0.39 ± 0.06	
baboon	0.49 ± 0.17	Meguro et al 1999
human	0.31 ± 0.02	Blin et al 1991

Table S7.
Temporal cortex glucose utilization rate in mammals.

Species	Glucose utilization ($\mu mol/g \cdot min$)	Reference
mouse	0.93 ± 0.10	Quelven et al 2004, estim
rat	1.08 ± 0.12	Waschke et al 1993, auditory
rat	0.94 ± 0.13	Dube et al 2001
rat	1.45 ± 0.04	Nehling et al 1988, auditory
rat	1.34 ± 0.11	Nehling et al 2000, auditory
rat	1.22 ± 0.08	Levant et al 2004
rat	1.34 ± 0.08	Zocchi et al 2001, auditory
average rat	1.23 ± 0.10	
rabbit	1.02 ± 0.10	Passero et al 1981
cat	0.97 ± 0.09	Schwartzman et al 1986, auditory
cat	1.18 ± 0.04	Sokoloff 1984, auditory
cat	1.05 ± 0.08	Lydic et al 1991
average cat	1.07 ± 0.08	
monkey	0.36 ± 0.07	Noda et al 2002
monkey	0.40 ± 0.02	Porrino et al 2002
monkey	0.79 ± 0.04	Kennedy et al 1978, auditory
average monkey	0.52 ± 0.04	
baboon	0.47 ± 0.14	Meguro et al 1999
human	0.23 ± 0.02	Blomqvist et al 1998
human	0.37 ± 0.03	Heiss et al 1985
human	0.31 ± 0.08	Haier et al 1992
human	0.36 ± 0.06	De Volder et al 1997
human	0.32 ± 0.03	Redies et al 1989
human	0.30 ± 0.06	Maquet et al 1992
average human	0.32 ± 0.05	

Table S8.
Sensorimotor cortex glucose utilization rate in mammals.

Species	Glucose utilization ($\mu mol/g \cdot min$)	Reference
mouse	1.21 ± 0.09	Bouilleret et al 2000
rat	0.84 ± 0.13	Dube et al 2001
rat	0.89 ± 0.07	Waschke et al 1993
rat	0.90 ± 0.07	Zocchi et al 2001
rat	0.90 ± 0.09	Nehlig et al 2000
average rat	0.88 ± 0.09	
rabbit	0.83 ± 0.07	Passero et al 1981
cat	0.64 ± 0.03	Sokoloff 1984
monkey	0.44 ± 0.03	Kennedy et al 1978
baboon	0.49 ± 0.08	Meguro et al 1999
human	0.42 ± 0.03	Heiss et al 1985
human	0.33 ± 0.03	Blin et al 1991
average human	0.38 ± 0.03	

Table S9.
Occipital cortex glucose utilization rate in mammals.

Species	Glucose utilization ($\mu mol/g \cdot min$)	Reference
rat	1.00 ± 0.07	Levant et al 2004
rat	0.83 ± 0.09	Blin et al 1991
average rat	0.92 ± 0.08	
cat	1.26 ± 0.10	Lydic et al 1991
monkey	0.40 ± 0.09	Noda et al 2002
baboon	0.33 ± 0.06	Meguro et al 1999
human	0.32 ± 0.03	Redies et al 1989
human	0.28 ± 0.02	Blomqvist et al 1998
human	0.29 ± 0.05	Maquet et al 1992
human	0.42 ± 0.12	Haier et al 1992
human	0.33 ± 0.04	Bline et al 1991
average human	0.33 ± 0.05	

Table S10.
Parietal cortex glucose utilization rate in mammals.

Species	Glucose utilization ($\mu mol/g \cdot min$)	Reference
rat	0.77 ± 0.08	Waschke et al 1993
rat	0.89 ± 0.11	Dube et al 2001
rat	0.96 ± 0.04	Levant et al 2004
average rat	0.87 ± 0.12	
rabbit	0.73 ± 0.08	Passero et al 1981
cat	0.69 ± 0.08	Sokoloff 1984
monkey	0.47 ± 0.04	Kennedy et al 1978
baboon	0.47 ± 0.10	Meguro et al 1999
human	0.27 ± 0.02	Blomqvist et al 1998
human	0.37 ± 0.02	Heiss et al 1985
human	0.43 ± 0.15	Haier et al 1992
human	0.33 ± 0.04	Blin et al 1991
average human	0.35 ± 0.05	

Table S11.
Cerebral cortex average glucose utilization rate in mammals.
Data for Fig. 2E in the main text.

Species	Glucose utilization ($\mu mol/g \cdot min$)	Number of areas
mouse	1.10 ± 0.11	N=6
rat	0.95 ± 0.11	N=8
rabbit	0.84 ± 0.09	N=4
cat	0.86 ± 0.08	N=6
monkey	0.46 ± 0.06	N=8
baboon	0.46 ± 0.13	N=6
human	0.34 ± 0.05	N=8

Table S12.
Cerebral cortex average glucose utilization rate in mammals.
Data for Fig. S1 in the Supplementary Information.

Species	Glucose utilization ($\mu mol/g \cdot min$)	Number of areas	Reference
mouse	1.07 ± 0.10	N=5	Bouilleret et al 2000
rat	0.97 ± 0.04	N=8	Nehling et al 1988
rat	0.83 ± 0.13	N=10	Dube et al 2001
rat	0.88 ± 0.06	N=9	Levant et al 2004
rat	0.95 ± 0.13	N=7	Zocchi et al 2001
rat	0.88 ± 0.10	N=5	Waschke et al 1993
average rat	0.90 ± 0.09		
rabbit	0.83 ± 0.08	N=5	Passero et al 1981
cat	0.78 ± 0.12	N=5	Chugani et al 1991
cat	0.87 ± 0.06	N=4	Sokoloff 1984
cat	0.79 ± 0.04	N=5	Schwartzman et al 1986
average cat	0.81 ± 0.11		
monkey	0.43 ± 0.03	N=11	Porrino et al 2002
monkey	0.51 ± 0.03	N=10	Palombo et al 1990
monkey	0.37 ± 0.08	N=3	Noda et al 2002
monkey	0.57 ± 0.03	N=4	Kennedy et al 1978
average monkey	0.47 ± 0.04		
baboon	0.46 ± 0.10	N=6	Meguro et al 1999
human	0.37 ± 0.07	N=4	De Volder et al 1997
human	0.26 ± 0.02	N=4	Blomqvist et al 1998
human	0.39 ± 0.03	N=9	Heiss et al 1985
human	0.33 ± 0.04	N=6	Blin et al 1991
average human	0.34 ± 0.04		

Table S13.
Thalamus glucose utilization rate in mammals.

Species	Glucose utilization ($\mu mol/g \cdot min$)	Reference
mouse	1.19 ± 0.10	Bouilleret et al 2000
mouse	1.03 ± 0.10	Quelven et al 2004, estim.
average mouse	1.11 ± 0.10	
rat	0.92 ± 0.03	Nehling et al 1988
rat	0.88 ± 0.08	Nehling et al 2000
rat	0.94 ± 0.14	Dube et al 2001
rat	0.98 ± 0.05	Levant et al 2004
rat	0.72 ± 0.09	Waschke et al 1993
average rat	0.89 ± 0.09	
rabbit	0.90 ± 0.05	Passero et al 1981
cat	0.81 ± 0.11	Schwartzman et al 1986
cat	0.85 ± 0.15	Chugani et al 1991
cat	0.86 ± 0.08	Sokoloff 1984
average cat	0.84 ± 0.12	
monkey	0.41 ± 0.04	Porrino et al 2002
monkey	0.47 ± 0.02	Kennedy et al 1978
monkey	0.46 ± 0.03	Palombo et al 1990
average monkey	0.45 ± 0.03	
baboon	0.51 ± 0.16	Meguro et al 1999
human	0.39 ± 0.05	De Volder et al 1997
human	0.27 ± 0.02	Blomqvist et al 1998
human	0.36 ± 0.03	Heiss et al 1985
human	0.32 ± 0.04	Blin et al 1991
human	0.35 ± 0.05	Maquet et al 1992
average human	0.34 ± 0.04	

Table S14.
Cerebellum glucose utilization rate in mammals.

Species	Glucose utilization ($\mu mol/g \cdot min$)	Reference
mouse	0.98 ± 0.10	Quelven et al 2004, estim.
rat	0.61 ± 0.02	Nehling et al 1988
rat	0.68 ± 0.08	Dube et al 2001
rat	0.57 ± 0.12	Waschke et al 1993
average rat	0.62 ± 0.07	
cat	0.71 ± 0.08	Sokoloff 1984
cat	0.65 ± 0.13	Chugani et al 1991
average cat	0.68 ± 0.11	
monkey	0.38 ± 0.02	Kennedy et al 1978
monkey	0.27 ± 0.06	Noda et al 2002
monkey	0.46 ± 0.03	Palombo et al 1990
average monkey	0.37 ± 0.03	
baboon	0.32 ± 0.05	Meguro et al 1999
human	0.33 ± 0.05	De Volder et al 1997
human	0.28 ± 0.02	Blomqvist et al 1998
human	0.32 ± 0.06	Heiss et al 1985
human	0.29 ± 0.03	Blin et al 1991
human	0.24 ± 0.07	Maquet et al 1992
average human	0.29 ± 0.05	

Table S15.
Hypothalamus glucose utilization rate in mammals.

Species	Glucose utilization ($\mu mol/g \cdot min$)	Reference
mouse	0.59 ± 0.14	Bouilleret et al 2000
mouse	0.56 ± 0.10	Quelven et al 2004, estim.
average mouse	0.58 ± 0.12	
rat	0.48 ± 0.02	Nehling et al 1988
rat	0.49 ± 0.09	Dube et al 2001
rat	0.44 ± 0.03	Levant et al 2004
rat	0.39 ± 0.05	Waschke et al 1993
rat	0.54 ± 0.05	Nehling et al 2000
rat	0.40 ± 0.04	Blin et al 1991
average rat	0.46 ± 0.05	
rabbit	0.62 ± 0.03	Passero et al 1981
cat	0.41 ± 0.06	Chugani et al 1991
cat	0.41 ± 0.04	Sokoloff 1984
average cat	0.41 ± 0.05	
monkey	0.30 ± 0.03	Porrino et al 2002
monkey	0.26 ± 0.04	Kennedy et al 1978
average monkey	0.28 ± 0.04	
human	0.27 ± 0.02	Blin et al 1991
Mammilary body:		
mouse	1.56 ± 0.13	Bouilleret et al 2000
rat	1.03 ± 0.04	Nehling et al 1988
rat	1.04 ± 0.17	Dube et al 2001
rat	0.76 ± 0.05	Waschke et al 1993
average rat	0.94 ± 0.09	
rabbit	0.68 ± 0.03	Passero et al 1981
cat	0.99 ± 0.32	Chugani et al 1991
cat	0.85 ± 0.03	Sokoloff 1984
average cat	0.92 ± 0.18	
monkey	0.57 ± 0.06	Palombo et al 1990
monkey	0.57 ± 0.03	Kennedy et al 1978
average monkey	0.57 ± 0.05	

Table S16.
Hippocampus glucose utilization rate in mammals.

Species	Glucose utilization ($\mu mol/g \cdot min$)	Reference
mouse	0.84 ± 0.13	Bouilleret et al 2000, CA1+CA3
rat	0.60 ± 0.05	Waschke et al 1993, CA1+CA3
rat	0.63 ± 0.08	Dube et al 2001, CA1+CA3
rat	0.64 ± 0.03	Nehling et al 1988, dorsal + ventral
rat	0.56 ± 0.03	Levant et al 2004
rat	0.62 ± 0.03	Zocchi et al 2001, CA1+CA3
average rat	0.61 ± 0.04	
rabbit	0.58 ± 0.03	Passero et al 1981
cat	0.52 ± 0.15	Chugani et al 1991, dorsal + ventral
cat	0.54 ± 0.04	Lydic et al 1991
cat	0.57 ± 0.05	Sokoloff 1984
average cat	0.54 ± 0.10	
monkey	0.32 ± 0.05	Noda et al 2002
monkey	0.37 ± 0.02	Porrino et al 2002
monkey	0.33 ± 0.01	Palombo et al 1990
monkey	0.39 ± 0.02	Kennedy et al 1978
average monkey	0.35 ± 0.03	
baboon	0.32 ± 0.07	Meguro et al 1999
human	0.37 ± 0.05	Heiss et al 1985
human	0.23 ± 0.07	Maquet et al 1992
human	0.23 ± 0.03	Blin et al 1991
average human	0.28 ± 0.05	

Table S17.
Amygdala glucose utilization rate in mammals.

Species	Glucose utilization ($\mu mol/g \cdot min$)	Reference
mouse	0.58 ± 0.10	Bouilleret et al 2000
rat	0.67 ± 0.07	Dube et al 2001
rat	0.52 ± 0.02	Nehling et al 1988
rat	0.44 ± 0.03	Levant et al 2004
rat	0.57 ± 0.06	Nehling et al 2000
rat	0.40 ± 0.07	Waschke et al 1993
average rat	0.52 ± 0.05	
rabbit	0.39 ± 0.01	Passero et al 1981
cat	0.54 ± 0.06	Chugani et al 1991
cat	0.34 ± 0.04	Sokoloff 1984
average cat	0.44 ± 0.05	
monkey	0.33 ± 0.01	Porrino et al 2002
monkey	0.38 ± 0.02	Palombo et al 1990
monkey	0.25 ± 0.02	Kennedy et al 1978
average monkey	0.32 ± 0.02	
human	0.21 ± 0.03	Blin et al 1991

Table S18.
Septum glucose utilization rate in mammals.

Species	Glucose utilization ($\mu mol/g \cdot min$)	Reference
mouse	0.67 ± 0.12	Bouilleret et al 2000
mouse	0.79 ± 0.03	Quelven et al 2004, estim.
average mouse	0.73 ± 0.08	
rat	0.48 ± 0.03	Waschke et al 1993
rat	0.47 ± 0.08	Dube et al 2001
rat	0.61 ± 0.02	Nehling et al 1988
rat	0.45 ± 0.05	Nehling et al 2000
rat	0.45 ± 0.03	Levant et al 2004
rat	0.61 ± 0.07	Zocchi et al 2001
average rat	0.51 ± 0.05	
rabbit	0.48 ± 0.02	Passero et al 1981
cat	0.37 ± 0.05	Chugani et al 1991
cat	0.39 ± 0.03	Sokoloff 1984
average cat	0.38 ± 0.04	
monkey	0.30 ± 0.03	Porrino et al 2002
monkey	0.27 ± 0.01	Palombo 1990
monkey	0.26 ± 0.02	Kennedy 1978
average monkey	0.28 ± 0.02	

Table S19.
Caudate glucose utilization rate in mammals.

Species	Glucose utilization ($\mu mol/g \cdot min$)	Reference
mouse	1.25 ± 0.16	Bouilleret et al 2000
rat	0.85 ± 0.04	Nehling et al 1988
rat	0.82 ± 0.14	Dube et al 2001
rat	0.82 ± 0.06	Waschke et al 1993
rat	0.85 ± 0.09	Nehling et al 2000
rat	0.77 ± 0.09	Blin et al 1991
rat	0.87 ± 0.07	Zocchi et al 2001
average rat	0.83 ± 0.09	
cat	0.77 ± 0.06	Schwartzman et al 1986
cat	0.83 ± 0.11	Chugani et al 1991
cat	0.77 ± 0.02	Sokoloff 1984
average cat	0.79 ± 0.10	
monkey	0.60 ± 0.04	Porrino et al 2002
monkey	0.52 ± 0.03	Kennedy et al 1978
monkey	0.51 ± 0.03	Palombo et al 1990
average monkey	0.54 ± 0.04	
baboon	0.41 ± 0.08	Meguro et al 1999
human	0.32 ± 0.06	Blin et al 1991
human	0.39 ± 0.04	Heiss et al 1985
human	0.35 ± 0.06	Maquet et al 1992
average human	0.35 ± 0.06	

Table S20.
Substantia nigra glucose utilization rate in mammals.

Species	Glucose utilization ($\mu mol/g \cdot min$)	Reference
mouse	0.88 ± 0.07	Bouilleret et al 2000
rat	0.57 ± 0.01	Nehling et al 1988
rat	0.52 ± 0.08	Waschke et al 1993
rat	0.56 ± 0.04	Nehling et al 2000
rat	0.52 ± 0.03	Levant et al 2004
rat	0.49 ± 0.09	Blin et al 1991
average rat	0.53 ± 0.04	
rabbit	0.48 ± 0.03	Passero et al 1981
cat	0.34 ± 0.03	Schwartzman et al 1986
cat	0.46 ± 0.06	Chugani et al 1991
cat	0.40 ± 0.05	Sokoloff et al 1984
average cat	0.40 ± 0.05	
monkey	0.44 ± 0.01	Porrino et al 2002
monkey	0.29 ± 0.03	Kennedy et al 1978
monkey	0.37 ± 0.01	Palombo et al 1990
average monkey	0.37 ± 0.02	
human	0.26 ± 0.05	Blin et al 1991

Table S21.
Globus pallidus glucose utilization rate in mammals.

Species	Glucose utilization ($\mu mol/g \cdot min$)	Reference
mouse	0.76 ± 0.03	Bouilleret et al 2000
rat	0.46 ± 0.02	Nehling et al 1988
rat	0.42 ± 0.04	Dube et al 2001
rat	0.45 ± 0.05	Waschke et al 1993
rat	0.44 ± 0.06	Nehling et al 2000
rat	0.57 ± 0.05	Zocchi et al 2001
rat	0.44 ± 0.04	Levant et al 2004
average rat	0.46 ± 0.04	
rabbit	0.42 ± 0.02	Passero et al 1981
cat	0.38 ± 0.04	Schwartzman et al 1986
cat	0.44 ± 0.10	Chugani et al 1991
cat	0.36 ± 0.02	Sokoloff 1984
average cat	0.39 ± 0.05	
monkey	0.31 ± 0.04	Porrino et al 2002
monkey	0.26 ± 0.02	Kennedy et al 1978
monkey	0.27 ± 0.02	Palombo et al 1990
average monkey	0.28 ± 0.03	

Table S22.
Brain stem glucose utilization rate in mammals.

Species	Glucose utilization ($\mu mol/g \cdot min$)	Reference
Superior colliculus:		
mouse	0.93 ± 0.08	Bouilleret et al 2000
mouse	0.87 ± 0.10	Quelven et al 2004, estim.
average mouse	0.90 ± 0.09	
rat	0.76 ± 0.03	Nehling et al 1988
rat	0.67 ± 0.13	Dube et al 2001
rat	0.71 ± 0.05	Waschke et al 1993
rat	0.73 ± 0.07	Levant et al 2004
average rat	0.72 ± 0.08	
rabbit	0.62 ± 0.01	Passero et al 1981
cat	0.86 ± 0.20	Chugani et al 1991
cat	0.82 ± 0.08	Sokoloff 1984
average cat	0.84 ± 0.14	
monkey	0.55 ± 0.04	Kennedy et al 1978
monkey	0.55 ± 0.03	Palombo et al 1990
average monkey	0.55 ± 0.04	
Inferior colliculus:		
mouse	0.99 ± 0.05	Quelven et al 2004, estim.
rat	1.64 ± 0.05	Nehling et al 1988
rat	1.70 ± 0.15	Nehling et al 2000
rat	1.14 ± 0.22	Dube et al 2001
rat	1.27 ± 0.25	Waschke et al 1993
rat	1.71 ± 0.05	Levant et al 2004
average rat	1.49 ± 0.20	
rabbit	1.26 ± 0.03	Passero et al 1981
cat	2.03 ± 0.74	Chugani et al 1991
cat	1.71 ± 0.20	Sokoloff 1984
average cat	1.87 ± 0.50	
monkey	1.03 ± 0.06	Kennedy et al 1978
monkey	1.30 ± 0.05	Palombo et al 1990
average monkey	1.17 ± 0.06	

Table S23.
White matter glucose utilization rate in mammals.

Species	Glucose utilization ($\mu mol/g \cdot min$)	Reference
Corpus callosum		
mouse	0.47 ± 0.10	Quelven et al 2004, estim.
rat	0.29 ± 0.03	Waschke et al 1993
rat	0.22 ± 0.02	Nehlig et al 2000
average rat	0.26 ± 0.03	
cat	0.15 ± 0.02	Sokoloff 1984
cat	0.22 ± 0.02	Chugani et al 1991
average cat	0.19 ± 0.02	
monkey	0.11 ± 0.00	Kennedy et al 1978
monkey	0.11 ± 0.01	Palombo et al 1990
average monkey	0.11 ± 0.01	
Internal capsule		
rat	0.25 ± 0.04	Waschke et al 1993
rat	0.32 ± 0.03	Nehlig et al 1988
average rat	0.29 ± 0.04	
rabbit	0.26 ± 0.03	Passero et al 1981
cat	0.17 ± 0.02	Sokoloff et al 1984
monkey	0.13 ± 0.01	Kennedy et al 1978
monkey	0.11 ± 0.02	Palombo et al 1990
average monkey	0.12 ± 0.02	

3. **Supplementary Methods**.

In the above studies CMR_{glc} was measured by [^{14}C]2-deoxyglucose (2DG) technique (Sokoloff et al, 1977) (all species except baboon and human) or its non-invasive modification [^{18}F]fluoro-2-deoxy-D-glucose (FDG) (Phelps et al, 1979) (baboon, human, and one case of macaque (Noda et al, 2002)) under similar standard physiological conditions (Tables S2-S23). Thus the glucose data are directly

comparable. The measurements of CMR$_{O_2}$ (Table S1) were performed either by the Ketty-Schmidt method (Ketty and Schmidt, 1948) (rat, sheep, macaque, baboon, human) or by other less-known techniques (cat, dog). However, a removal of cat and dog from the scaling plot in Fig. 1A does not change the oxygen metabolic exponent.

The values of glucose utilization for mouse taken from Quelven et al (2004) are estimates. These authors provide only ratios of glucose utilization in relation to corpus callosum. I obtained the absolute values by taking piriform cortex glucose utilization, which is recorded both in Quelven et al (2004) and in Bouilleret et al (2000), as a reference frame.

Data for brain volumes were taken either directly from the source or from standard sources (Stephan et al 1981; Hofman 1985):
Mouse: 0.35 cm^3, rat: 2.26 cm^3, squirrel: 7.6 cm^3, rabbit: 11.5 cm^3, cat: 31.8 cm^3, dog: 72.0 cm^3, macaque monkey: 100.0 cm^3, sheep: 114.0 cm^3, goat: 117.0 cm^3, baboon: 137.0 cm^3, human: 1389.0 cm^3.

References

Blin J., et al., *Brain Res.* **568**, 215-222 (1991).
Blomqvist G., et al., *Acta Physiol. Scand.* **163**, 403-415 (1998).
Bouilleret V., et al., *Brain Res.* **852**, 255-262 (2000).
Chugani H.T., et al., *J. Cereb. Blood Flow Metabol.* **11**, 35-47 (1991).
Clarke D.D. and Sokoloff L., In *Basic Neurochemistry.* Eds: Siegel G.J., et al. (Raven Press, New York, 1994), pp. 645-680.
De Volder A.G., et al., *Brain Res.* **750**, 235-244 (1997).
Dube C., et al., *Exper. Neurol.* **167**, 227-241 (2001).
Frerichs K.U., et al., *Am. J. Physiol.* **268**, R445-R453 (1995).
Haier R.J., et al., *Brain Res.* **570**, 134-143 (1992).
Heiss W.-D., et al., *Brain Res.* **327**, 362-366 (1985).
Herdman S.J., et al., *J. Neurosci.* **9**, 1150-1162 (1989).
Hofman M.A., *Brain Behav. Evol.* **32**, 17-26 (1988).
Kennedy C., et al., *Ann. Neurol* **4**, 293-301 (1978).
Levant B. and Pazdernik T.L., *Brain Res.* **1003**, 159-167 (2004).

Linde R., et al., *Acta Physiol. Scand.* **165**, 395-401 (1999).
Lydic R., et al., *J. Comp. Neurol.* **304**, 517-529 (1991).
Madsen P.L., et al., *J. Appl. Physiol.* **70**, 2597-2601 (1991).
Meguro K., et al., *Brain* **122**, 1519-1531 (1999).
Nakanishi O. et al., *Can. J. Anaesth.* **44**, 1008-1013 (1997).
Nehlig A., et al., *J. Neurosci.* **8**, 2321-2333 (1988).
Nehlig A. and Boyet S., *Brain Res.* **858**, 71-77 (2000).
Nemoto E.M., et al., *J. Neurosurg. Anesthesiol.* **6**, 170-174 (1994).
Noda A., et al., *Brain Res.* **936**, 76-81 (2002).
Maquet P., et al., *Brain Res.* **571**, 149-153 (1992).
Palombo E., et al., *J. Neurosci.* **10**, 860-869 (1990).
Passero S., et al., *Neurosci. Lett.* **21**, 345-349 (1981).
Pell J.M. and Bergman E.N., *Am. J. Physiol.* **244**, E282-E289 (1983).
Pelligrino D.A., et al., *Am. J. Physiol.* **268**, R276-R283 (1987).
Porrino L.J., et al., *J. Neurosci.* **22**, 7687-7694 (2002).
Quelven I., et al., *Neurosci.* **126**, 441-449 (2004).
Redies C., et al., *Am. J. Physiol.* **256**, E805-E810 (1989).
Sahlin C., et al., *Brain Res.* **403**, 313-332 (1987).
Schwartzman R.J., et al., *Brain Res.* **398**, 113-120 (1986).
Sokoloff L., *Metabolic Probes of Central Nervous System Activity in Experimental Animals and Man*, (Sinauer, Sunderland, MA, 1984).
Stephan, H., Baron, G., and Frahm, H.D. New and revised data on volumes of brain structure in insectivores and primates. *Folia Primatol.* **35**, 1-29 (1981).
Stingele R., et al., *Am. J. Physiol.* **271**, H579-H587 (1996).
Waschke K., et al., *Am. J. Physiol.* **265**, H1243-H1248 (1993)
Zocchi A., et al., *Neurosci. Lett.* **306**, 177-180 (2001).

Methods for measuring metabolism can be found in:

Ketty S.S. and Schmidt C.F., *J. Clin. Invest.* **27**, 476-483 (1948).
Phelps M.E. et al, *Ann. Neurol.* **6**, 371-388 (1979).
Sokoloff L. et al., *J. Neurochem.* **28**, 897-916 (1977).
